# Systematic Review of Reported HIV Outbreaks, Pakistan, 2000–2019

**DOI:** 10.3201/eid2704.204205

**Published:** 2021-04

**Authors:** Elizabeth M. Rabold, Hammad Ali, Danielle Fernandez, Martha Knuth, Karl Schenkel, Rana Jawad Asghar, Mirza Amir Baig, Saqib Shaikh, Oliver Morgan

**Affiliations:** Centers for Disease Control and Prevention, Atlanta, Georgia, USA (E.M. Rabold, H. Ali, D. Fernandez, M. Knuth);; World Health Organization, Geneva, Switzerland (K. Schenkel, O. Morgan);; Global Health Strategists and Implementers, Karachi, Pakistan (R.J. Asghar);; Pakistan Field Epidemiology and Laboratory Training Program, Karachi (M.A. Baig);; Sindh AIDS Control Program, Larkana, Pakistan (S. Shaikh)

**Keywords:** HIV, outbreaks, Pakistan, iatrogenic infection, infection prevention and control, blood safety, systematic review, viruses, bloodborne pathogens, sexually transmitted infections

## Abstract

Unsafe injection practices and injection drug use have been linked to multiple HIV outbreaks in Pakistan since 2003; however, few studies have systematically analyzed the causes of these outbreaks. We conducted a systematic review of published English-language literature indexed in bibliographic databases and search engines and a focused gray literature review to collate and analyze all reported HIV outbreaks in Pakistan during 2000–2019. Of 774 unique publications reviewed, we identified 25 eligible publications describing 7 outbreaks. More than half occurred during 2016–2019. The primary sources of transmission were iatrogenic transmission, affecting children, persons with chronic medical conditions, and the general population (4 outbreaks); injection drug use (2 outbreaks); and a combination of both (1 outbreak). In the absence of robust HIV testing and surveillance in Pakistan, timely and detailed outbreak reporting is important to understand the epidemiology of HIV in the country.

## Abstract 2

In the absence of robust testing programs, timely and detailed outbreak reporting is essential for HIV control.

The first cases of HIV in Pakistan were reported in 1987, with epidemiologic evidence supporting the importation of cases by migrant workers from the Gulf States (*1*–*3*). Since that time, noncontinuous surveillance assessments have noted high prevalence of HIV in certain populations; the most recent 2016–2017 prevalence estimates were 38.4% among persons who inject drugs (PWID), 7.2% among transgender persons, and 5.6% among men who have sex with men (*4*–*8*). By comparison, the prevalence in the general population is 0.1%, representing ≈190,000 persons living with HIV (PLHIV), including 6,100 children <15 years of age, according to 2019 Joint United Nations Programme on HIV/AIDS (UNAIDS) estimates (*8*,*9*). Approximately 44,758 (24%) PLHIV were registered with the National AIDS Control Programme with a known diagnosis as of December 2020, and of these, only 24,362 (54%) were receiving antiretroviral therapy (ART) (*10*). These statistics are far below the UNAIDS 90–90–90 HIV treatment targets (90% of HIV-positive persons being aware of their status; of those, 90% receiving ART; and of those, 90% being virally suppressed) aimed at controlling the AIDS epidemic; most PLHIV (87%) in Pakistan are not receiving treatment (*11*).

In April 2019, a major HIV outbreak in Larkana District in Pakistan was identified by local and provincial public health officials (*12*). After several ill children with HIV-negative parents tested positive for HIV, the provincial Sindh AIDS Control Program began a voluntary district-wide testing campaign. During April 25–June 28, 2019, a total of 30,192 persons were tested for HIV; 876 (2.9%) were HIV positive, and 82% of those were children <15 years of age. A World Health Organization (WHO) report cited unsafe medical practices and poor infection control programs as key risk factors for infection (*12*) and noted that this outbreak was the fourth HIV outbreak in Larkana since 2003. A cursory review of the literature, however, did not identify peer-reviewed publications on all of these referenced outbreaks. The objective of our systematic review was to identify and collate data from all reported HIV outbreaks in Pakistan to describe overarching themes and aid in future prevention efforts.

## Methods

We followed the PRISMA statement and the Cochrane Handbook to conduct this systematic review (Appendix Table 1) (*13*). We searched Medline, Embase, CAB Abstracts, Global Health, PsycInfo, Cochrane Library, Scopus, Academic Search Complete, Cumulative Index to Nursing and Allied Health Literature, ProQuest Central, PubMed Central, Virtual Health Library, and Google Scholar to identify English-language publications on reported HIV outbreaks in Pakistan during January 1, 2000–December 31, 2019. We limited the search to studies published after January 1, 2000, because the earliest reported HIV outbreak in Pakistan occurred in 2003 (*14*). To complement the published literature search, we conducted a comprehensive search of the gray literature (i.e., publications not published in indexed peer-reviewed journals), including UNAIDS reports, WHO reports, and International AIDS Society conference abstracts. In addition, we manually reviewed Pakistan’s provincial and national Ministry of Health websites. The following search strategy was used for database and gray literature searches: (HIV or AIDS, any associated synonyms, or both) AND (outbreak, epidemic, pandemic, or cluster) AND (Pakistan [or all subnational units]). We omitted location criteria for manual review of Pakistan governmental websites. The full search strategy is detailed in Appendix Table 2. We used Endnote X9 (Clarivate Analytics, https://endnote.com) to import and manage retrieved records. To identify duplicate reports, we used the EndNote automated “find duplicates” function, with preferences set to match by title, author, and year; a second round of manual de-duplication was performed by using the same matching criteria. We grouped the remaining reports by database, search engine, and source, and authors reviewed these independently. We used a shared database to track the progress of the reviews.

We systematically screened and reviewed results from the published and gray literature search (Figure). We screened titles and abstracts, and we defaulted to reviewing the abstract if the title had an unclear focus and reviewing the full report if no abstract was available, counting it among the number of abstracts reviewed. We included publications that reported data on outbreaks of HIV or sudden increases in cases in Pakistan. For the purpose of this systematic review, we defined an outbreak as an unexpected number of HIV cases identified through targeted testing or key population surveillance, labeled and reported as an outbreak, and leading to an evaluation or investigation. We excluded abstracts without published final reports (unless identified in the gray literature), reports that provided prevalence or incidence data only (including key population surveys), opinion pieces without mention of a specific outbreak, mathematical modeling studies, epidemiologic analyses, reports without quantitative data, and preprint reports. We also excluded reports where the author did not define the described cases as an outbreak or did not provide a discrete geographic, temporal, or epidemiologic link. If identical reports were published in >1 journal, the earliest publication was included. Similarly, if identical or nearly identical reports were published in a journal and also included as a conference abstract, we included only the published report. If a report included outbreak data as well as a subset of data in a case control, cohort, or cross-sectional investigation, we included data on the larger outbreak and the study. We reviewed journal submission guidelines to determine whether a publication was peer-reviewed.

**Figure:**
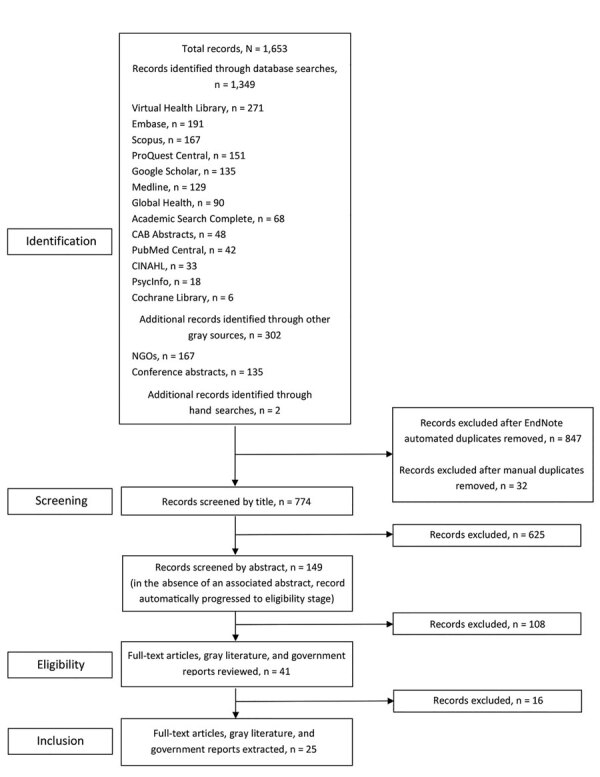
Identification and selection of studies reporting HIV outbreaks in Pakistan, January 2000–September 2019. CINAHL, Cumulative Index to Nursing and Allied Health Literature; NGOs, nongovernmental organizations.

We organized eligible publications, gray literature, and government reports by geographic location and year of the reported outbreak. We included reports describing multiple outbreaks under each appropriate outbreak heading. We extracted year and type of report, investigating agency and source or reference for primary data, number of persons tested and diagnosed with HIV, case positivity rate (defined as the percentage of persons positive among the number tested within the period defined by the authors of the publication), notable demographic and behavioral characteristics of case-patients, major risk factors, and other relevant information (Appendix Table 3). We noted instances where articles used media reports as their primary citation. One author independently reviewed initial data extraction of all eligible reports for concurrence. If necessary, we reached out to corresponding authors of individual reports for clarification.

## Results

Our initial search identified 1,653 records published during January 2000–December 2019. We removed 879 (53%) duplicate reports identified across multiple databases or search engines through automated and manual processes (Figure). Of the remaining 774 de-duplicated reports, 625 (81%) were excluded after review of the title and 108 (14%) were excluded after review of the abstract. We excluded 16 reports upon review of the full article, gray literature, or government report, leaving 25 (3%) reports eligible for inclusion.

The 25 reports identified by our search strategy described 7 outbreaks: 4 in Punjab Province (Sargodha, Sargodha District [2007]; Kot Imrana, Sargodha District [2018]; Jalalpur Jattan, Gujrat District [2008]; and Faisalabad, Faisalabad District [2019]) and 3 in Sindh Province (Larkana, Larkana District [2003 and 2016] and Ratodero, Larkana District [2019]) (Appendix Table 3). Six (24%) reports described >2 outbreaks. 

Case-positivity rates ranged from 1.3% to 51.8%, varying in part because of sampling methods. The potential source of 4 of the 7 outbreaks was reported as iatrogenic transmission through unsafe healthcare practices at clinics, hospitals, and dialysis centers; 2 outbreaks were attributed to injection drug use, and 1 outbreak was attributed to both. Several reports described a potential association with unqualified healthcare providers (frequently designated as quacks in Pakistan [*15*]), in general, or with a specific provider. Some reports also reported cultural practices as a contributing factor to transmission. Populations most affected by the outbreaks varied by proposed etiology; iatrogenic causes affected the general community, including women and children, as well as persons living with specific medical conditions, such as end-stage renal disease. Recreational drug use affected primarily PWID, most frequently men.

Our review identified 5 reports in peer-reviewed literature, with the remaining reports published as letters to the editor or correspondence, nongovernmental organization and government reports, and conference abstracts. National or provincial AIDS control programs led the initial investigations of 4 of the 7 outbreaks; the National Institutes of Health-Pakistan and Field Epidemiology Training Program–Pakistan and district health departments provided data for the other 3 outbreaks. The Ratodero (2019) outbreak had additional support from WHO, other United Nations agencies, local universities, and other international and local partners. Of the 25 reports, 17 (68%) describe this single outbreak. Other outbreaks had more limited data, often limited to case counts and affected population. Authors were often not directly affiliated with the primary data but rather briefly described testing statistics, demographic information, and risk factors obtained from investigations from government entities, media reports, and other sources. Some discrepancies were noted across reports pertaining to the same outbreak, and many reports did not provide complete information on case-positivity rates, study period, or method of data collection. Authors occasionally (4 [16%]) used media reports as the primary source of information. Though most outbreaks had at least 1 article citing primary data or data directly from a testing program, the single report found for the Faisalabad (2019) outbreak cited only a newspaper article. Of the 25 reports describing the 7 outbreaks, only 5 reports provided detailed outbreak investigation information. Despite more extensive investigations, these reports still had limited ability to draw conclusions or conduct statistical comparisons because of study design (e.g., no comparison group [*16*] or small sample size [*17*]). Only 1 of the 25 reports, describing an outbreak investigation in Jalalpar Jattan (2008), included phylogenetic information (*16*), which demonstrated that transmission likely occurred over a decade, reflecting endemic disease rather than an outbreak.

## Discussion

Our review identified 25 reports describing 7 HIV outbreaks during 2000–2019 in Pakistan: 3 in Sindh Province and 4 in Punjab Province. Of these, 4 were identified during 2016–2019. In 2019, two outbreaks were reported: a large outbreak primarily affecting children in Ratodero in Larkana, a district with multiple prior outbreaks, and an outbreak in Faisalabad, primarily infecting PWID. Case-positivity rates ranged from 1.3% to 51.8%, and populations most affected varied by outbreak but included PWID; persons living with specific medical conditions; and the general population, including women and children. The level of detail pertaining to the description of data collection and investigation methods varied across the publications, and much of the data provided were collected not by authors but by national, provincial, and district health departments and other government entities. Iatrogenic transmission (57%), injection drug use (29%), or both (14%) were identified as the potential sources of the outbreaks; no outbreak solely attributable to sexual transmission was reported.

Iatrogenic transmission from unsafe healthcare practices and poor infection prevention and control was identified as the primary or contributing risk factor in 5 of the 7 HIV outbreaks (Jalalpur Jattan [2008], Kot Imrana [2018], Larkana [2016], Ratodero [2019], and Faisalabad [2019]). From a recent survey in Pakistan, researchers estimated that ≈38% of surveyed physicians likely reused syringes (*18*). Data from the latest Demographic Health Survey indicate that ≈9% of injections given to patients in Pakistan are unsafe, and every person receives an average of 4.1 therapeutic injections per year in Pakistan (*19*). Extrapolating from this frequency and safety data, approximately 1 in 3 persons might receive an unsafe injection every year in Pakistan (*19*). Furthermore, cross-sectional studies of persons with thalassemia in Pakistan have shown a high prevalence of bloodborne infections, including HIV, hepatitis B, and hepatitis C, suggestive of infection from blood transfusions (*20*,*21*). Nosocomial or iatrogenic transmission including unsafe blood transfusions and reuse of medical equipment has contributed to several HIV outbreaks in other countries, including ≈10,000 children in orphanages in Romania (*22*), >400 children in Libya with frequent co-infection with hepatitis B and C (*23*), and 242 adults and children in Cambodia (*24*).

Several factors might play a role in the propagation of unsafe injection practices in low-income countries. These factors include sociocultural factors such as healthcare providers’ belief that compliance is better with injections than with oral medication and patients might seek healthcare elsewhere if not provided injections; financial incentives on the part of both patient and provider through fee-for-injection practices and contingent on provider ability to purchase and maintain a supply of injecting equipment; corruption, when money allocated for healthcare, such as disposable injecting equipment, is used elsewhere, leading to reuse of equipment; lack of policies and procedures around safe injection practices, such that policies forbidding the reuse of injecting equipment are not implemented nor enforced in low-income countries as they are in high-income countries; ready access to injectable medications without a prescription; and lack of awareness of risks associated with unsafe injection practices (*25*). Given these factors, developing a multi-strategy approach that might be adapted and tailored as necessary might help prevent future outbreaks of HIV and other bloodborne pathogens. These strategies include community and healthcare provider education to address excessive and unnecessary use of therapeutic injections, implementation and monitoring of policies around single-use injecting equipment, and addressing gaps in infection prevention and control.

Injection drug use was reported as the primary or contributing cause of HIV transmission in 3 of the 7 outbreaks (Larkana [2003], Sargodha [2007], and Faisalabad [2019]). Periodic HIV surveillance data are available for key populations in specific cities from the National AIDS Control Programme Integrated Biologic and Behavioral Surveillance surveys, but they are not designed to measure prevalence for the general population or key populations in rural areas (*4*–*8*). The HIV prevalence among PWID documented by each survey increased from 10.8% to 38.4%; however, because the survey was expanded to new cities across the different reporting periods, direct comparison of the change in prevalence is not possible. Whether any change in prevalence might be attributable to sporadic outbreaks or a steady increase in HIV prevalence in this subpopulation is unknown. None of the literature describing outbreaks with injection drug use as the primary or contributing source of transmission reported a phylogenetic analysis, leaving timelines of infections in these outbreaks unclear.

Although the Integrated Biologic and Behavioral Surveillance surveys offer insight into HIV prevalence among key populations, the absence of routine HIV surveillance in the general population prevents understanding of the actual burden of the HIV epidemic in Pakistan. Considering the high prevalence of HIV in PWID, men who have sex with men, and female sex workers, as well as unsafe injection practices as we have described, spillover to the general population is only a matter of time. Widespread surveillance of HIV might be challenging and might yield little information given the low general population prevalence of 0.1% (*9*). However, adding surveillance of targeted populations at higher risk, such as pregnant women, patients at infectious disease or tuberculosis clinics, and persons requiring frequent transfusions, might provide early warning signs to changes in HIV prevalence. Likewise, systematic monitoring of the blood supply might represent an efficient, less costly approach to surveillance. Currently, routine surveillance is not conducted in any of these settings.Although phylogenetic analyses, which assist in understanding circulating strains and subtypes, might contribute to our understanding of a rise in cases, only 1 publication identified in this review reported a phylogenetic analysis; it showed that, despite preliminary data suggestive of a new outbreak, transmission occurred over a decade (*16*). Without comprehensive surveillance and phylogenetic data, ascertaining whether new HIV diagnoses or a sudden increase in diagnoses in an area represent an outbreak or simply missed HIV diagnoses with endemic transmission over time is difficult.

Outbreaks are underrepresented in the literature; those that are published have limited ability to characterize the full epidemiologic and phylogenetic footprint of an outbreak. Nonsystematic tracking of media reports identified at least 2 other potential outbreaks known to national or provincial AIDS control programs but not described by our systematic review (*26*,*27*). Given the frequency of media reports of HIV outbreaks, albeit without full epidemiologic data, and well-documented data on the widespread prevalence of unsafe injections across Pakistan, the paucity of systematic outbreak investigations is striking. Of the reports included in our systematic review, only 5 (20%) were peer-reviewed; the remaining were published as letters to the editor, editorials, general correspondence, abstracts, nongovernment organization publications, and government reports, without clear description of methods, study design, and data collection. Given the limited outbreak investigations and robust data reporting in peer-reviewed and gray literature, our systematic review likely underestimates the frequency of the problem and its associated burden of disease.

The main strength of our review is that we searched multiple bibliographic databases, with the addition of Google Scholar and the Virtual Health Library, nongovernmental organization and government websites, and conference abstracts to ensure all relevant publications were captured. However, we note several limitations. First, we recognize that the definition of an outbreak is challenging in the setting of limited phylogenetic and surveillance data. A study by Ansari et al. (*16*) determined that the observed increase in cases was likely a progression of endemic disease only after the results of phylogenetic analysis. As such, other outbreaks reported in this review might, if the same analyses were available, have been determined not to be outbreaks. Second, our literature review was limited to English-language publications. Although a potential exists for missing articles written in Urdu and other local languages, English is one of the official languages in the country and is the predominant language for scientific and medical research dissemination in Pakistan (*28**,**29*). Finally, although unlikely, a small chance exists that a unique outbreak might have been mentioned in a publication focusing on surveillance or other data and thus been missed by our tiered review approach. We also recognize that outbreak reports written by government entities might be for internal review only or might be posted online for a limited time, resulting in a possible bias towards availability of more recent outbreaks.

In summary, reported outbreaks in Pakistan suggest that the spread of HIV might continue if adequate prevention strategies are not adopted. Education campaigns to improve knowledge in the general public about unsafe injection practices, both therapeutic and recreational, might limit HIV transmission and occurrence of outbreaks. Assessing patient and provider misconceptions about the benefits of therapeutic injections could guide public health messaging and reduce demand for unnecessary medical interventions. Reviewing injection safety and infection prevention and control practices could inform healthcare reform efforts to limit iatrogenic exposures and the potential for HIV outbreaks. Last, developing and putting into place comprehensive HIV surveillance systems could assist in outbreak identification, prompting investigations that explore risk factors and underlying transmission sources. Reporting of outbreaks in peer-reviewed literature, including epidemiologic studies and phylogenetic analyses, might shed additional light on the etiologies of outbreaks and effective prevention strategies. Across the spectrum of reports identified by our systematic review, all reports had the consistent message of sounding an alarm and highlighting a potentially rapidly growing problem in Pakistan.

Appendix Additional information about systematic review of reported HIV outbreaks, Pakistan, 2000–2019.
