# Supplementary material for: Systematic Review of Reported HIV Outbreaks, Pakistan, 2000–2019
Source: Emerg Infect Dis. 2021 Apr;27(4):1039–47. doi: 10.3201/eid2704.204205 (PMC8007288; doi:10.3201/eid2704.204205)
Supplement: Appendix — Additional information about systematic review of reported HIV outbreaks, Pakistan, 2000–2019. [file 20-4205-Techapp-s1.pdf]

Article DOI: https://doi.org/10.3201/eid2704.204205

Systematic Review of Reported HIV Outbreaks, Pakistan, 2000–2019

Appendix

Table of Contents:

Appendix Table 1. PRISMA 2009 checklist

Appendix Table 2. Literature Search Strategy

Appendix Table 3. HIV Outbreaks in Pakistan, January 2000–December 2019

Appendix Table 1. PRISMA 2009 checklist*

Article section	Item no.	Checklist item	Reported on page no.
Title	1	Identify the report as a systematic review, meta-analysis, or both.	p. 1
Abstract			
Structured summary	2	Provide a structured summary including, as applicable: background; objectives; data sources; study eligibility criteria, participants, and interventions; study appraisal and synthesis methods; results; limitations; conclusions and implications of key findings; systematic review registration number.	Summary, p. 2
Introduction			
Rationale	3	Describe the rationale for the review in the context of what is already known.	Introduction, pp. 2-3
Objectives	4	Provide an explicit statement of questions being addressed with reference to participants, interventions, comparisons, outcomes, and study design (PICOS).	Introduction, p. 3
Methods			
Protocol and registration	5	Indicate if a review protocol exists, if and where it can be accessed (e.g., Web address), and, if available, provide registration information including registration number.	N/A
Eligibility criteria	6	Specify study characteristics (e.g., PICOS, length of follow-up) and report characteristics (e.g., years considered, language, publication status) used as criteria for eligibility, giving rationale.	Methods, p. 3
Information sources	7	Describe all information sources (e.g., databases with dates of coverage, contact with study authors to identify additional studies) in the search and date last searched.	Methods, pp. 3-4
Search	8	Present full electronic search strategy for at least one database, including any limits used, such that it could be repeated.	Methods, p. 3, Appendix Table 1,
Study selection	9	State the process for selecting studies (i.e., screening, eligibility, included in systematic review, and, if applicable, included in the meta-analysis).	Methods, p. 4
Data collection process	10	Describe method of data extraction from reports (e.g., piloted forms, independently, in duplicate) and any processes for obtaining and confirming data from investigators.	Methods, pp. 3-4
Data items	11	List and define all variables for which data were sought (e.g., PICOS, funding sources) and any assumptions and simplifications made.	Methods, p. 4
Risk of bias in individual studies	12	Describe methods used for assessing risk of bias of individual studies (including specification of whether this was done at the study or outcome level), and how this information is to be used in any data synthesis.	N/A
Summary measures	13	State the principal summary measures (e.g., risk ratio, difference in means).	N/A
Synthesis of results	14	Describe the methods of handling data and combining results of studies, if done, including measures of consistency (e.g., I2) for each meta-analysis.	N/A
Risk of bias across studies	15	Specify any assessment of risk of bias that may affect the cumulative evidence (e.g., publication bias, selective reporting within studies).	Methods, p. 4
Additional analyses	16	Describe methods of additional analyses (e.g., sensitivity or subgroup analyses, meta-regression), if done, indicating which were pre-specified.	N/A
Results			
Study selection	17	Give numbers of studies screened, assessed for eligibility, and included in the review, with reasons for exclusions at each stage, ideally with a flow diagram.	Results, p. 5, Figure
Study characteristics	18	For each study, present characteristics for which data were extracted (e.g., study size, PICOS, follow-up period) and provide the citations.	Results, pp. 5-6, Appendix Table 3
Risk of bias within studies	19	Present data on risk of bias of each study and, if available, any outcome level assessment (see item 12).	Results, pp. 5-6
Results of individual studies	20	For all outcomes considered (benefits or harms), present, for each study: (a) simple summary data for each intervention group (b) effect estimates and confidence intervals, ideally with a forest plot.	N/A
Synthesis of results	21	Present results of each meta-analysis done, including confidence intervals and measures of consistency.	N/A
Risk of bias across studies	22	Present results of any assessment of risk of bias across studies (see Item 15).	Results, pp. 5-6
Additional analysis	23	Give results of additional analyses, if done (e.g., sensitivity or subgroup analyses, meta-regression [see Item 16]).	N/A
Discussion			
Summary of evidence	24	Summarize the main findings including the strength of evidence for each main outcome; consider their relevance to key groups (e.g., healthcare providers, users, and policy makers).	Discussion, pp. 6-10

Article section	Item no.	Checklist item	Reported on page no.
Limitations	25	Discuss limitations at study and outcome level (e.g., risk of bias), and at review-level (e.g., incomplete retrieval of identified research, reporting bias).	Discussion, pp. 9-10
Conclusions	26	Provide a general interpretation of the results in the context of other evidence, and implications for future research.	Discussion, pp. 7-10
Funding	27	Describe sources of funding for the systematic review and other support (e.g., supply of data); role of funders for the systematic review.	p. 10

*Adapted from Moher D, Liberati A, Tetzlaff J, Altman DG, The PRISMA Group. Preferred reporting items for systematic reviews and meta-analyses: the PRISMA Statement. PLoS Med 6:e1000097 (https://doi.org/10.1371/journal.pmed.1000097). Additional information available at https://www.prisma-statement.org.

Appendix Table 2. Literature Search Strategy

Database or entity	Search Strategy*
Search request #1: HIV/AIDS outbreaks in Pakistan. Searches for literature on the subject. Limits: English, 2000–2019. Original search run date: 09/20/2019. Updated search run date: 12/11/20.	
Medline (Ovid) 1946–present	(<br>((hiv OR human immunodeficiency virus* OR acquired immune deficiency syndrome* OR acquired immunodeficiency syndrome*) AND (case OR cases)).ti.<br>OR<br>(<br>(hiv OR human immunodeficiency virus* OR acquired immune deficiency syndrome* OR acquired immunodeficiency syndrome*).mp. OR exp HIV/ OR exp Acquired Immunodeficiency Syndrome/<br>AND<br>(outbreak* OR epidemic* OR pandemic* OR cluster*).mp. OR exp Disease Outbreaks/<br>))<br>AND<br>(Pakistan* OR azad Kashmir OR baolchistan OR Bahawalpur OR dera ghazi khan OR Faisalabad OR Gilgit OR Gujranwala OR Gujrat OR Islamabad* OR Jhelum OR karachi OR Khyber Pakhtunkhwa OR kot imrana OR Lahore OR Larkana OR mardan OR multan OR Muzaffarabad OR Nowshera OR Peshawar OR Punjab OR quetta OR Rahim yar khan OR Rawalpindi OR Sargodha OR Sheikhupura OR Sialkot OR Sindh OR sukkur).mp. OR exp Pakistan/
Embase (Ovid) 1947–present	(<br>((hiv OR human immunodeficiency virus* OR acquired immune deficiency syndrome* OR acquired immunodeficiency syndrome*) AND (case OR cases)).ti.<br>OR<br>(<br>(hiv OR human immunodeficiency virus* OR acquired immune deficiency syndrome* OR acquired immunodeficiency syndrome*).mp. OR exp Human immunodeficiency virus/ OR exp acquired immune deficiency syndrome/<br>AND<br>(outbreak* OR epidemic* OR pandemic* OR cluster*).mp. OR exp epidemic/<br>))<br>AND<br>(Pakistan* OR azad Kashmir OR baolchistan OR Bahawalpur OR dera ghazi khan OR Faisalabad OR Gilgit OR Gujranwala OR Gujrat OR Islamabad* OR Jhelum OR karachi OR Khyber Pakhtunkhwa OR kot imrana OR Lahore OR Larkana OR mardan OR multan OR Muzaffarabad OR Nowshera OR Peshawar OR Punjab OR quetta OR Rahim yar khan OR Rawalpindi OR Sargodha OR Sheikhupura OR Sialkot OR Sindh OR sukkur).mp. OR exp Pakistan/
CAB Abstracts (Ovid) 1910–present	(<br>((hiv OR human immunodeficiency virus* OR acquired immune deficiency syndrome* OR acquired immunodeficiency syndrome*) AND (case OR cases)).ti.<br>OR<br>(<br>(hiv OR human immunodeficiency virus* OR acquired immune deficiency syndrome* OR acquired immunodeficiency syndrome*).mp. OR exp human immunodeficiency viruses/ OR exp acquired immune deficiency syndrome/<br>AND<br>(outbreak* OR epidemic* OR pandemic* OR cluster*).mp. OR exp epidemics/ OR exp outbreaks/<br>))<br>AND<br>(Pakistan* OR azad Kashmir OR baolchistan OR Bahawalpur OR dera ghazi khan OR Faisalabad OR Gilgit OR Gujranwala OR Gujrat OR Islamabad* OR Jhelum OR karachi OR Khyber Pakhtunkhwa OR kot imrana OR Lahore OR Larkana OR mardan OR multan OR Muzaffarabad OR Nowshera OR Peshawar OR Punjab OR quetta OR Rahim yar khan OR Rawalpindi OR Sargodha OR Sheikhupura OR Sialkot OR Sindh OR sukkur).mp. OR exp pakistan/
Global Health (Ovid) 1910–present	(<br>((hiv OR human immunodeficiency virus* OR acquired immune deficiency syndrome* OR acquired immunodeficiency syndrome*) AND (case OR cases)).ti.<br>OR

Database or entity	Search Strategy*
PsycInfo (Ovid) 1806–present	((hiv OR human immunodeficiency virus* OR acquired immune deficiency syndrome* OR acquired immunodeficiency syndrome*).mp. OR exp human immunodeficiency viruses/ OR exp acquired immune deficiency syndrome/ AND (outbreak* OR epidemic* OR pandemic* OR cluster*).mp. OR exp epidemics/ OR exp outbreaks/)) AND (Pakistan* OR azad Kashmir OR baolchistan OR Bahawalpur OR dera ghazi khan OR Faisalabad OR Gilgit OR Gujranwala OR Gujrat OR Islamabad* OR Jhelum OR karachi OR Khyber Pakhtunkhwa OR kot imrana OR Lahore OR Larkana OR mardan OR multan OR Muzaffarabad OR Nowshera OR Peshawar OR Punjab OR quetta OR Rahim yar khan OR Rawalpindi OR Sargodha OR Sheikhupura OR Sialkot OR Sindh OR sukkur).mp. OR exp pakistan/ (((hiv OR human immunodeficiency virus* OR acquired immune deficiency syndrome* OR acquired immunodeficiency syndrome*) AND (case OR cases)).ti. OR ((hiv OR human immunodeficiency virus* OR acquired immune deficiency syndrome* OR acquired immunodeficiency syndrome*).mp. OR exp HIV/ OR exp AIDS/ AND (outbreak* OR epidemic* OR pandemic* OR cluster*).mp. OR exp Epidemics/)) AND (Pakistan* OR azad Kashmir OR baolchistan OR Bahawalpur OR dera ghazi khan OR Faisalabad OR Gilgit OR Gujranwala OR Gujrat OR Islamabad* OR Jhelum OR karachi OR Khyber Pakhtunkhwa OR kot imrana OR Lahore OR Larkana OR mardan OR multan OR Muzaffarabad OR Nowshera OR Peshawar OR Punjab OR quetta OR Rahim yar khan OR Rawalpindi OR Sargodha OR Sheikhupura OR Sialkot OR Sindh OR sukkur).mp.
Cochrane Library	#1(hiv OR “human immunodeficiency virus” OR “acquired immune deficiency syndrome” OR “acquired immunodeficiency syndrome”):ti AND (case OR cases):ti Limits 110<br>#2(hiv OR “human immunodeficiency virus” OR “acquired immune deficiency syndrome” OR “acquired immunodeficiency syndrome”):ti,ab,kw Limits 25455<br>#3MeSH descriptor: [HIV] explode all treesMeSH 2972<br>#4MeSH descriptor: [Acquired Immunodeficiency Syndrome] explode all trees MeSH 1732<br>#5#2 OR #3 OR #4Limits 25455<br>#6(Pakistan* OR “azad Kashmir” OR baolchistan OR Bahawalpur OR “dera ghazi khan” OR Faisalabad OR Gilgit OR Gujranwala OR Gujrat OR Islamabad* OR Jhelum OR karachi OR “Khyber Pakhtunkhwa” OR “kot imrana” OR Lahore OR Larkana OR mardan OR multan OR Muzaffarabad OR Nowshera OR Peshawar OR Punjab OR quetta OR “Rahim yar khan” OR Rawalpindi OR Sargodha OR Sheikhupura OR Sialkot OR Sindh OR sukkur):ti,ab,kwLimits 2578<br>#7MeSH descriptor: [Pakistan] explode all treesMeSH 413<br>#8#6 OR #7Limits 2578<br>#9(outbreak* OR epidemic* OR pandemic* OR cluster*):ti,ab,kwLimits 25990<br>#10MeSH descriptor: [Disease Outbreaks] explode all treesMeSH 270<br>#11#9 OR #10Limits 25990<br>#12#5 AND #11Limits 1560<br>#13#1 OR #12Limits 1659<br>#14#13 AND #8Limits 6
Scopus 1960–present	((TITLE (hiv OR “human immunodeficiency virus” OR “acquired immune deficiency syndrome” OR “acquired immunodeficiency syndrome”) AND TITLE (case OR cases)) OR ((TITLE-ABS-KEY (hiv OR “human immunodeficiency virus” OR “acquired immune deficiency syndrome” OR “acquired immunodeficiency syndrome”)) AND (TITLE-ABS-KEY (outbreak* OR epidemic* OR pandemic* OR cluster*)))) AND (TITLE-ABS-KEY (pakistan* OR “azad Kashmir” OR baolchistan OR bahawalpur OR “dera ghazi khan” OR faisalabad OR gilgit OR gujranwala OR gujrat OR islamabad* OR jhelum OR karachi OR “Khyber Pakhtunkhwa” OR “kot imrana” OR lahore OR larkana OR mardan OR multan OR muzaffarabad OR nowshera OR peshawar OR punjab OR quetta OR “Rahim yar khan” OR rawalpindi OR sargodha OR sheikhupura OR sialkot OR sindh OR sukkur)) AND (LIMIT-TO (PUBYEAR, 2019) OR LIMIT-TO (PUBYEAR, 2018) OR LIMIT-TO (PUBYEAR, 2017) OR LIMIT-TO (PUBYEAR, 2016) OR LIMIT-TO (PUBYEAR, 2015) OR LIMIT-TO (PUBYEAR,

Database or entity	Search Strategy*
	2014) OR LIMIT-TO (PUBYEAR, 2013) OR LIMIT-TO (PUBYEAR, 2012) OR LIMIT-TO (PUBYEAR, 2011) OR LIMIT-TO (PUBYEAR, 2010) OR LIMIT-TO (PUBYEAR, 2009) OR LIMIT-TO (PUBYEAR, 2008) OR LIMIT-TO (PUBYEAR, 2007) OR LIMIT-TO (PUBYEAR, 2006) OR LIMIT-TO (PUBYEAR, 2005) OR LIMIT-TO (PUBYEAR, 2004) OR LIMIT-TO (PUBYEAR, 2003) OR LIMIT-TO (PUBYEAR, 2002) OR LIMIT-TO (PUBYEAR, 2001) OR LIMIT-TO (PUBYEAR, 2000)) AND (LIMIT-TO (LANGUAGE, “English”))
Academic Search Complete (Ebsco)	(<br>TI((hiv OR “human immunodeficiency virus” OR “acquired immune deficiency syndrome” OR “acquired immunodeficiency syndrome”) AND (case OR cases))<br>OR<br>(<br>TI,AB,KW,SU (hiv OR “human immunodeficiency virus” OR “acquired immune deficiency syndrome” OR “acquired immunodeficiency syndrome”)<br>AND<br>TI,AB,KW,SU (outbreak* OR epidemic* OR pandemic* OR cluster*)<br>))<br>AND<br>TI,AB,KW,SU (pakistan* OR “azad Kashmir” OR baolchistan OR bahawalpur OR “dera ghazi khan” OR faisalabad OR gilgit OR gujranwala OR gujrat OR islamabad* OR jhelum OR karachi OR “Khyber Pakhtunkhwa” OR “kot imrana” OR lahore OR larkana OR mardan OR multan OR muzaffarabad OR nowshera OR peshawar OR punjab OR quetta OR “Rahim yar khan” OR rawalpindi OR sargodha OR sheikhupura OR sialkot OR sindh OR sukkur)<br>Limits: peer-reviewed
CINAHL (Ebsco)	(<br>TI((hiv OR “human immunodeficiency virus” OR “acquired immune deficiency syndrome” OR “acquired immunodeficiency syndrome”) AND (case OR cases))<br>OR<br>(<br>TI,AB,SU (hiv OR “human immunodeficiency virus” OR “acquired immune deficiency syndrome” OR “acquired immunodeficiency syndrome”) OR (MH “Human Immunodeficiency Virus+”) OR (MH “Acquired Immunodeficiency Syndrome”)<br>AND<br>TI,AB,SU (outbreak* OR epidemic* OR pandemic* OR cluster*) OR (MH “Disease Outbreaks”)<br>))<br>AND<br>TI,AB,SU (pakistan* OR “azad Kashmir” OR baolchistan OR bahawalpur OR “dera ghazi khan” OR faisalabad OR gilgit OR gujranwala OR gujrat OR islamabad* OR jhelum OR karachi OR “Khyber Pakhtunkhwa” OR “kot imrana” OR lahore OR larkana OR mardan OR multan OR muzaffarabad OR nowshera OR peshawar OR punjab OR quetta OR “Rahim yar khan” OR rawalpindi OR sargodha OR sheikhupura OR sialkot OR sindh OR sukkur) OR (MH “Pakistan”)<br>Limits: peer-reviewed
ProQuest Central (Proquest) 1952–present	(<br>TI((hiv OR “human immunodeficiency virus” OR “acquired immune deficiency syndrome” OR “acquired immunodeficiency syndrome”) AND (case OR cases))<br>OR<br>(<br>TI,AB,SU (hiv OR “human immunodeficiency virus” OR “acquired immune deficiency syndrome” OR “acquired immunodeficiency syndrome”)<br>AND<br>TI,AB,SU (outbreak* OR epidemic* OR pandemic* OR cluster*)<br>))<br>AND<br>TI,AB,SU (pakistan* OR “azad Kashmir” OR baolchistan OR bahawalpur OR “dera ghazi khan” OR faisalabad OR gilgit OR gujranwala OR gujrat OR islamabad* OR jhelum OR karachi OR “Khyber Pakhtunkhwa” OR “kot imrana” OR lahore OR larkana OR mardan OR multan OR muzaffarabad OR nowshera OR peshawar OR punjab OR quetta OR “Rahim yar khan” OR rawalpindi OR sargodha OR sheikhupura OR sialkot OR sindh OR sukkur)<br>Limits: peer-reviewed

Database or entity	Search Strategy*
PubMed Central (NLM)	((((((((((hiv [Title] OR “human immunodeficiency virus” [Title] OR “acquired immune deficiency syndrome” [Title] OR “acquired immunodeficiency syndrome”[Title])) OR (hiv [Abstract] OR “human immunodeficiency virus” [Abstract] OR “acquired immune deficiency syndrome” [Abstract] OR “acquired immunodeficiency syndrome”[Abstract])) OR “hiv”[MeSH Terms]) OR “acquired immunodeficiency syndrome”[MeSH Terms])) AND ((((outbreak[Title] OR outbreaks[Title] OR epidemic[Title] OR epidemics[Title] OR pandemic[Title] OR pandemics[Title] OR cluster[Title] OR clusters[Title])) OR (outbreak[Abstract] OR outbreaks[Abstract] OR epidemic[Abstract] OR epidemics[Abstract] OR pandemic[Abstract] OR pandemics[Abstract] OR cluster[Abstract] OR clusters[Abstract])) OR “disease outbreaks”[MeSH Terms]))) OR (((hiv [Title] OR “human immunodeficiency virus” [Title] OR “acquired immune deficiency syndrome” [Title] OR “acquired immunodeficiency syndrome”[Title])) AND (case[Title] OR cases[Title])))) AND ((((pakistan* [Title] OR “azad Kashmir” [Title] OR baolchistan [Title] OR bahawalpur [Title] OR “dera ghazi khan” [Title] OR faisalabad [Title] OR gilgit [Title] OR gujranwala [Title] OR gujrat [Title] OR islamabad [Title] OR jhelum [Title] OR karachi [Title] OR “Khyber Pakhtunkhwa” [Title] OR “kot imrana” [Title] OR lahore [Title] OR larkana [Title] OR mardan [Title] OR multan [Title] OR muzaffarabad [Title] OR nowshera [Title] OR peshawar [Title] OR punjab [Title] OR quetta [Title] OR “Rahim yar khan” [Title] OR rawalpindi [Title] OR sargodha [Title] OR sheikhupura [Title] OR sialkot [Title] OR sindh [Title] OR sukkur[Title])) OR (pakistan* [Abstract] OR “azad Kashmir” [Abstract] OR baolchistan [Abstract] OR bahawalpur [Abstract] OR “dera ghazi khan” [Abstract] OR faisalabad [Abstract] OR gilgit [Abstract] OR gujranwala [Abstract] OR gujrat [Abstract] OR islamabad [Abstract] OR jhelum [Abstract] OR karachi [Abstract] OR “Khyber Pakhtunkhwa” [Abstract] OR “kot imrana” [Abstract] OR lahore [Abstract] OR larkana [Abstract] OR mardan [Abstract] OR multan [Abstract] OR muzaffarabad [Abstract] OR nowshera [Abstract] OR peshawar [Abstract] OR punjab [Abstract] OR quetta [Abstract] OR “Rahim yar khan” [Abstract] OR rawalpindi [Abstract] OR sargodha [Abstract] OR sheikhupura [Abstract] OR sialkot [Abstract] OR sindh [Abstract] OR sukkur[Abstract])) OR “pakistan”[MeSH Terms])
Virtual Health Library (WHO)	(tw:(hiv OR “human immunodeficiency virus” OR “acquired immune deficiency syndrome” OR “acquired immunodeficiency syndrome”)) AND (tw:(pakistan* OR “azad Kashmir” OR baolchistan OR bahawalpur OR “dera ghazi khan” OR faisalabad OR gilgit OR gujranwala OR gujrat OR islamabad* OR jhelum OR karachi OR “Khyber Pakhtunkhwa” OR “kot imrana” OR lahore OR larkana OR mardan OR multan OR muzaffarabad OR nowshera OR peshawar OR punjab OR quetta OR “Rahim yar khan” OR rawalpindi OR sargodha OR sheikhupura OR sialkot OR sindh OR sukkur)) AND (tw:(outbreak* OR epidemic* OR pandemic* OR cluster*))<br>OR<br>(ti:(hiv OR “human immunodeficiency virus” OR “acquired immune deficiency syndrome” OR “acquired immunodeficiency syndrome”)) AND (ti:(case OR cases)) AND (tw:(pakistan* OR “azad Kashmir” OR baolchistan OR bahawalpur OR “dera ghazi khan” OR faisalabad OR gilgit OR gujranwala OR gujrat OR islamabad* OR jhelum OR karachi OR “Khyber Pakhtunkhwa” OR “kot imrana” OR lahore OR larkana OR mardan OR multan OR muzaffarabad OR nowshera OR peshawar OR punjab OR quetta OR “Rahim yar khan” OR rawalpindi OR sargodha OR sheikhupura OR sialkot OR sindh OR sukkur))
Google Scholar (narrow search using Harzing’s Publish or Perish)	(Outbreak OR cluster) AND Allintitle: Pakistan hiv<br>(Outbreak OR cluster) AND Allintitle: Pakistan “human immunodeficiency virus”<br>(Outbreak OR cluster) AND Allintitle: Pakistan AIDS<br>(Outbreak OR cluster) AND Allintitle: Pakistan “acquired immune deficiency syndrome”<br>(Outbreak OR cluster) AND Allintitle: Pakistan “acquired immunodeficiency syndrome”<br>Allintitle: Pakistan hiv epidemic<br>Allintitle: Pakistan hiv cases<br>Allintitle: Pakistan aids cases
Search request #2: HIV/AIDS outbreaks in Pakistan. Additional searches for gray literature on the subject. Limits: English, 2000–2019. Search date: 11/25/2020	
UNAIDS	Pakistan AND outbreak
WHO	(tw:(Pakistan* OR “azad Kashmir” OR baolchistan OR Bahawalpur OR “dera ghazi khan” OR Faisalabad OR Gilgit OR Gujranwala OR Gujrat OR Islamabad* OR Jhelum OR karachi OR “Khyber Pakhtunkhwa” OR “kot imrana” OR Lahore OR Larkana OR mardan OR multan OR Muzaffarabad OR Nowshera OR Peshawar OR Punjab OR quetta OR “Rahim yar khan” OR Rawalpindi OR Sargodha OR Sheikhupura OR Sialkot OR Sindh OR sukkur)) AND (tw:(hiv OR “human immunodeficiency virus” OR “acquired immune deficiency syndrome” OR “acquired immunodeficiency syndrome” OR AIDS)) AND (tw:(outbreak* OR epidemic* OR pandemic* OR cluster*))
IAS Conference	(Pakistan OR lahore) AND outbreak<br>http://programme.ias2019.org/Search/Search?search = pakistan<br>https://programme.aids2018.org/<br>https://www.ias2017.org/<br>https://www.aids2016.org/<br>http://www.abstract-archive.org/<br>About 384 abstracts on Pakistan

Database or entity	Search Strategy*
Search request #3: HIV/AIDS outbreaks in Pakistan. Additional hand searches of government Web sites for reports on the subject. Limits: English, 2000–2019. Search date: 12/11/2020	
Government of Balochistan Province	(HIV OR AIDS) AND (outbreak OR epidemic OR pandemic OR cluster) http://balochistan.gov.pk
Health Department, Government of Punjab Province	(HIV OR AIDS) AND (outbreak OR epidemic OR pandemic OR cluster) https://health.punjab.gov.pk/Index.aspx
Health Department, Government of Sindh	(HIV OR AIDS) AND (outbreak OR epidemic OR pandemic OR cluster) https://www.sindhhealth.gov.pk/
Government of Northwest Frontier Province	(HIV OR AIDS) AND (outbreak OR epidemic OR pandemic OR cluster) [no website found]
National AIDS Control Programme	(HIV OR AIDS) AND (outbreak OR epidemic OR pandemic OR cluster) https://nacp.gov.pk/
National Institute of Health-Islamic Republic of Pakistan	(HIV OR AIDS) AND (outbreak OR epidemic OR pandemic OR cluster) https://www.nih.org.pk/

*To identify duplicates, we used the EndNote automated “find duplicates” function with preference set to match title, author, and year.

Appendix Table 3. HIV outbreaks in Pakistan, January 2000– December 2019

Publication	Initial investigating agency	No. positive of no. tested (case positivity rate)	Primary population	Outbreak identification, transmission routes, and reported risk factors	Notes
Larkana, Larkana District, Sindh Province, 2003 (*1–3*)					
Shah et al, 2004*; Rehman et al, 2007†; Altaf et al, 2016*	SACP	17 positive among 175–183 tested (9.3%–9.7%)	PWID	Testing was initiated in PWID after identification of an index patient through routine surveillance. Rehman et al: In a case control study (7 cases and 14 controls), injection drug use in groups, syringe reuse, sex with commercial sex workers, >3 casual sexual partners, and early initiation of sex were identified as risk factors.	Shah et al: Authors reported that an epidemiologic study is forthcoming, but no report was identified in this literature review.‡ Rehman et al: It is unclear if the cases in the case control study belong to the original population of the 2003 outbreak, as the timing of the investigation relative to the outbreak was not reported.
Sargodha, Sargodha District, Punjab Province, 2007 (*4*)					
Emmanuel et al, 2009†	NIH-Pakistan	205 positive among 400 tested (51.3%)	PWID, all men	Testing was conducted as part of routine surveillance of PWID, with a multi-stage cluster sampling design (205 HIV-positive and 195 HIV-negative). Authors identified injecting in groups, sharing paraphernalia, and living in northern areas of the city as risk factors.	
Jalalpur Jattan, Gujrat District, Punjab Province, 2008 (*5–7*)					
Ansari et al, 2013†; Qadar et al, 2019*; Davlidova et al, 2019†	FELTP-NIH Pakistan performed the investigation after New Light AIDS Control Society conducted screening camps	88 positive among 246 tested in 2008 (35.8%)	General population, including women and children	Ansari et al: Community testing was initiated after an increase in diagnoses of HIV. Among 53 cases interviewed during the investigation, authors identified a high prevalence (96%) of therapeutic injections. Ear and nose piercings (women), surgical and dental procedures, and barber shop visits (men) were also common.	Ansari et al: 74 of 88 (84%) found positive on RDT were also positive on ELISA. Phylogenetic analysis indicated that infections were likely acquired over a decade and did not reflect an outbreak. Davlidova et al: Authors noted that an additional 750 people were diagnosed with HIV in 2018, potentially indicative of endemic transmission.
Larkana, Larkana District, Sindh Province, 2016 (*3*)					
Altaf et al, 2016*	NACP	56 positive among 205 tested (27.3%)	Patients with end-stage renal disease	Testing initiated at a dialysis center after identification of an index patient. Authors cited poor infection control practices and procedures and use of blood products from unregulated laboratories and blood banks as risk factors.	Positive cases were diagnosed by RDT; of a sample of 20 cases, 19 (95%) were later confirmed positive on laboratory testing.
Kot Imrana, Sargodha District, Punjab Province, 2018 (*6–12*)					
Zaid et al, 2018*§; Wahid, 2019*; Qadar et al, 2019*; Masood, 2019*; Lancet Infectious Diseases Editorial Team, 2019*; Davlidova et al, 2019†; Zahra et al, 2019*	District health department and PACP in Sargodha	35–204 positive among 2,717 town residents (1.3%–7.5% town prevalence); unknown number tested	General population, with higher prevalence in women and children	Community testing was initiated after several individuals received an HIV diagnosis in June 2018. Multiple authors reported that infected patients had received unsafe injections from one specific quack.¶ Zaid et al: Authors hypothesized that lack of HIV education, quacks,¶ use of contaminated syringes, re-use of razors at barber shops, unsafe sexual practices, including with sex workers, same-sex relations, and injection drug use were risk factors.	Wahid: Additional testing through 2019 revealed 669 positive among 5,000 tested (13.4%).
Ratodero, Larkana District, Sindh Province, 2019 (*7,10–24*)					

Publication	Initial investigating agency	No. positive of no. tested (case positivity rate)	Primary population	Outbreak identification, transmission routes, and reported risk factors	Notes
Arif, 2019*; Ahmed et al, 2019*§; Green, 2019*; Lancet Infectious Diseases Editorial Team, 2019*; Masood, 2019* Davlidova et al, 2019†; Zaid et al, 2019*§; Zahra et al, 2019*§; Dyer, 2019* Shaikh et al, 2019#; Mir, 2019#; WHO, 2019#; NIH Islamabad, 2019#; WHO, 2019#; UNAIDS, 2019#; UNAIDS, 2019#; NIH Islamabad, 2019#	SACP, with support from a WHO-led investigation, in partnership with SACP, Aga Khan University, Dow University, FELTP, UN agencies, and local partners	157–1,112 positive among 4,100–36,000 tested (3.1%–3.8%)	Approximately 80% of cases were children	Shaikh et al: 12 children with persistent fever received an HIV diagnosis in April 2019. Among 5 children with confirmatory testing at SACP Referral Lab, none had parents with HIV, prompting initiation of community testing. UNAIDS: Preliminary findings from the joint investigation concluded that poor infection control practices, including a lack of sterilization and the re-use of syringes and intravenous drips, were the most notable risk factors. Other reports also included unsafe practices at blood banks, improper hospital waste management, association with a specific physician, male circumcision with unhygienic blades, and ear and nose piercing with unsafe needles as potential risk factors.	Mir: Case control study of 406 pediatric cases and 406 controls is forthcoming.
Faisalabad, Faisalabad District, Punjab Province, 2019 (*12*)					
Zahra et al, 2019*§	PACP	2,863 positive among unknown number tested	PWID, located in the cities of Chiniot, Sahiwal, Jhang, and Nankana Sahib	First identification of positive patients was not reported. Authors reported that most individuals acquired HIV through reused syringes and needles, medical services from quacks,¶ and dental surgeries.	

PLHIV, People Living With HIV; SACP, Sindh AIDS Control Program; PWID, People Who Inject Drugs; NIH, National Institute of Health; FELTP, Field Epidemiology Laboratory Training Program; NACP, National AIDS Control Programme; WHO, World Health Organization; RDT, Rapid Diagnostic Test; PACP, Punjab AIDS Control Programme

*Letter to the editor, correspondence, editorial, or other non-peer-reviewed publication.

†Peer-reviewed publication.

‡Per correspondence with Rehman et al, the data from their 2007 report does not represent the forthcoming epidemiologic investigation of the Larkana, Larkana District, 2003 outbreak described by the Shah, 2004 report.

§Media report cited as primary reference.

¶A quack is an unqualified healthcare provider.

#Grey literature or government report.

References

1. Shah SA, Altaf A, Mujeeb SA, Memon A. An outbreak of HIV infection among injection drug users in a small town in Pakistan: potential for national implications. Int J STD AIDS. 2004;15:209. PubMed https://doi.org/10.1258/095646204322916713

2. ur Rehman N, Emmanuel F, Akhtar S. HIV transmission among drug users in Larkana, Pakistan. Trop Doct. 2007;37:58–9. https://doi.org/10.1258/004947507779952096

3. Altaf A, Pasha S, Vermund SH, Shah SA. A second major HIV outbreak in Larkana, Pakistan. J Pak Med Assoc. 2016;66:1510–1. PubMed

4. Emmanuel F, Archibald C, Razaque A, Sandstrom P. Factors associated with an explosive HIV epidemic among injecting drug users in Sargodha, Pakistan. J Acquir Immune Defic Syndr. 2009;51:85–90. PubMed https://doi.org/10.1097/QAI.0b013e31819906f3

5. Ansari JA, Salman M, Safdar RM, Ikram N, Mahmood T, Zaheer HA, et al. HIV/AIDS outbreak investigation in Jalalpur Jattan (JPJ), Gujrat, Pakistan. J Epidemiol Glob Health. 2013;3:261–8. PubMed https://doi.org/10.1016/j.jegh.2013.06.001

6. Qadar LT, Shabbir O, Asghar SA. An uprising threat in the Pakistani society—the HIV outbreak [letter]. J Pak Med Assoc. 2019;69:606. PubMed

7. Davlidova S, Abidi SH, Ali S. Healthcare malpractice and continuing HIV outbreaks in Pakistan. BMJ Glob Health. 2019;4:e001920.

8. Zaid M, Afzal MS. HIV outbreak in Pakistan. Lancet Infect Dis. 2018;18:601. PubMed https://doi.org/10.1016/S1473-3099(18)30281-0

9. Wahid B. An update on the severe outbreak of HIV in Kot Imrana, Pakistan. Lancet Infect Dis. 2019;19:241. PubMed https://doi.org/10.1016/S1473-3099(19)30038-6

10. Masood Z. Is Pakistan facing HIV epidemic? Journal of University Medical and Dental College. 2019;10:iii–iv [cited 2020 Dec 12]. https://www.jumdc.com/index.php/jumdc/issue/view/2

11. The Lancet Infectious Diseases. HIV epidemics in Pakistan. Lancet Infect Dis. 2019;19:671. PubMed https://doi.org/10.1016/S1473-3099(19)30316-0

12. Zahra A, Naveed M, Ahmad I, Saleem MA, Zeshan B. The syringes catastrophe of the HIV outbreak in Faisalabad, Punjab, Pakistan. Future Virol. 2019;14:571–2. https://doi.org/10.2217/fvl-2019-0084

13. Ahmed A, Hashmi FK, Khan GM. HIV outbreaks in Pakistan. Lancet HIV. 2019;6:e418. PubMed https://doi.org/10.1016/S2352-3018(19)30179-1

14. Arif F. HIV crisis in Sindh, Pakistan: the tip of the iceberg. Lancet Infect Dis. 2019;19:695–6. PubMed https://doi.org/10.1016/S1473-3099(19)30265-8

15. Green A. HIV epidemic in children in Pakistan raises concern. Lancet. 2019;393:2288. PubMed https://doi.org/10.1016/S0140-6736(19)31330-3

16. Zaid M, Ali M, Afzal MS. HIV outbreaks in Pakistan. Lancet HIV. 2019;6:e418–9. PubMed https://doi.org/10.1016/S2352-3018(19)30180-8

17. Dyer O. HIV: doctor who reused needles is blamed as nearly 900 children test positive in Pakistani city. BMJ. 2019;367:l6284. PubMed https://doi.org/10.1136/bmj.l6284

18. Shaikh S. Pakistan—HIV outbreak response [Conference presentation]. International AIDS Society 2019 Conference, Mexico City, Mexico [cited 2020 Dec 12]. http://programme.ias2019.org/Programme/Session/171

19. Mir F. Outbreak investigation and treatment challenges, Ratodero, District Larkana, Pakistan [Conference presentation]. International AIDS Society 2019 Conference, Mexico City, Mexico [cited 2020 Dec 12]. http://programme.ias2019.org/Programme/Session/171

20. National Institute of Health Islamabad. Seasonal awareness and alert letter, 45th issue, Islamabad 2019 [cited 2020 Dec 12]. https://www.nih.org.pk/wp-content/uploads/2019/07/45th-Issue-SAAL-final.pdf

21. National Institute of Health Islamabad. Seasonal awareness and alert letter, 44th issue, Islamabad 2019 [cited 2020 Dec 12]. https://www.nih.org.pk/wp-content/uploads/2019/03/44th-Issue-SAAL-final.pdf

22. World Health Organization. Public health round-up. Bull World Health Organ. 2019;97:517–8. PubMed https://doi.org/10.2471/BLT.19.010819

23. Joint United Nations Programme on HIV/AIDS (UNAIDS). UNAIDS continues to support the response to the HIV outbreak in Pakistan. Geneva: UNAIDS; 2019 [cited 2020 Dec 12]. https://www.unaids.org/en/resources/presscentre/featurestories/2019/june/20190618_pakistan

24. Joint United Nations Programme on HIV/AIDS (UNAIDS). Responding to the HIV outbreak in Larkana. Geneva: UNAIDS; 2019 [cited 2020 Dec 12]. https://www.unaids.org/en/resources/presscentre/featurestories/2019/june/20190611_larnaka_outbreak
